# Current Concepts in Artificial Intelligence-Assisted Arthroplasty: A Review of the Perioperative Pathway

**DOI:** 10.7759/cureus.99946

**Published:** 2025-12-23

**Authors:** Abdelrahman Sayed, Ahmed Elkohail, Ali Soffar, Mohamed Elbanna, Larisa Radu, Mohamed Wasim Shaffe Ahamed, Rhia Shah

**Affiliations:** 1 Trauma and Orthopaedics, Cardiff University Hospital, Cardiff, GBR; 2 Trauma and Orthopaedics, Princess Royal University Hospital, King's College NHS Foundation Trust, London, GBR; 3 Trauma and Orthopaedics, Epsom and St Helier University Hospitals, London, GBR

**Keywords:** ai, arthroplasty, artificial intelligence, orthopaedic, perioperative care

## Abstract

Artificial intelligence (AI) in arthroplasty care enhances preoperative planning, intraoperative preparation, and postoperative management across hip and knee replacement pathways. Preoperatively, AI recognizes implant designs on plain radiographs, supports revision planning, and can estimate the hip joint center, improving measurements and logistics for complex cases. Clinically, models assist with patient selection and risk stratification, helping forecast readmission or reoperation and guiding resource use. Postoperatively, supervised and ensemble learning predict complications, pain trajectories, dissatisfaction, attainment of minimally clinically important differences in patient-reported outcome measures (PROMs), and even prolonged opioid use, enabling earlier, personalized interventions. Reported benefits include faster, more accurate implant identification for revision surgery and tailored care pathways that can reduce time and cost in typically older, comorbid populations. However, widespread adoption requires rigorous external validation, transparent performance reporting, and reproducible evaluation standards to ensure models are calibrated, transportable, and safe across settings. Overall, the evidence indicates that integrating AI with clinical and imaging data can streamline decisions from preoperative risk assessment to postoperative follow-up, while highlighting the need for stronger multicenter studies, data standards, and governance to translate promising results into reliable, equitable clinical tools.

## Introduction and background

Healthcare utilizes artificial intelligence (AI) to develop predictive tools that aid clinicians in making complex decisions. Machine learning (ML), a specific type of AI, has found applications across various medical fields, including oncology, neurology, and orthopedic surgery [1-4]. Over the past few decades, the use of total hip arthroplasty (THA) has increased in parallel with life expectancy. Patients 65 years of age or older have seen a 16% increase in THA; this same group has also grown by 12%. Some European nations and Australia are expected to see a 95%-120% increase in THA between 2015 and 2050 [5-9].

By utilizing the predictive capabilities of AI within the context of THA, it is possible to achieve significant improvements in clinical decision-making. This, in turn, leads to a streamlined process that effectively reduces the time, cost, and overall complexity associated with THA procedures [10]. By facilitating more precise clinical prediction and decision-making during perioperative treatment, the incorporation of AI and ML technology into THA can advance the field of orthopaedics [11-13].

Early use of AI/ML techniques is possible in patient selection, planning, and risk prediction for readmission and reoperation. In particular, during revision arthroplasty preoperative planning, neural networks, a subset of ML, can detect failing implants [4]. After surgery, AI tools can forecast potential complications, patient-reported outcomes, pain levels, and even extended opioid use [14,15].

## Review

Overview of AI in orthopedic practice

John McCarthy initially proposed the idea of AI in 1956. At first, AI was seen as a theoretical development that would eventually allow computers to "learn." Through the use of algorithms to identify patterns, this learning process would entail carrying out automated activities with little to no direct human participation [14,15].

AI allows computers to quickly analyze vast amounts of data, a task impossible for humans. These computers use programmed algorithms to perform specific tasks such as categorizing, identifying, or linking variables, with the ability to assign different levels of importance to certain factors [16].

Computers are programmed with detailed instructions to identify specific characteristics. Their ability to do so accurately is frequently evaluated against a recognized benchmark, traditionally set by humans. AI systems are fed vast amounts of data, enabling them to quickly and consistently recognize patterns, usually in relation to a predefined result or series of results. System accuracy can be improved by adjusting the algorithm to facilitate more precise data assessment. The new result is then re-evaluated against the benchmark, its accuracy determined, and error correction measures integrated back into the original algorithm. This iterative process essentially "teaches" the AI system to become more precise and accurate, similar to how humans progressively learn through repeated exposure and review [17].

The process of "teaching" a computer system to autonomously or semi-autonomously execute a specific task is fundamentally known as "machine learning." This involves exposing the computer to large datasets, allowing it to identify patterns, make predictions, and refine its performance over time without explicit programming for each outcome [16,18-20].

AI algorithms are employed to identify arthroplasty components from X-rays by training them with imaging features that differentiate implant types or brands. These features include length, size, proximal body-to-stem proportions, curvature changes, neck-shaft angles, and collar presence. The AI system is initially provided with a training set of X-ray images of implants with known brands and models. It then analyzes these images, attempting to match radiographic features to known implant parameters. The system's accuracy is subsequently assessed by comparing its results to the correct answers. If inaccuracies occur, additional information is manually incorporated into the algorithm, and the training process is repeated. This iterative process continues, with more information being added, until an acceptable level of accuracy is achieved, at which point the AI system is ready for practical application [21,22].

AI can offer many benefits in daily orthopedic practice, including improvements in diagnosis, decision-making, surgical technique, and administration. Before surgery, AI can enhance the accuracy or speed of key diagnostic steps, such as identifying and classifying pathologies or determining implant types. The strong capability of AI in digital image analysis makes it well suited for these tasks. This is particularly useful for revision surgery, where correctly identifying existing implants, including older models, is crucial for planning salvage options and ordering necessary equipment [23,24].

Benefits of AI in arthroplasty

AI/ML-powered predictive models offer several advantages in hip and knee arthroplasty. They help surgeons manage costs by predicting the necessity of surgery, which is particularly beneficial for an often older and comorbid patient population. These tools assist providers in developing and refining personalized treatment plans by accurately identifying previous implants for revision surgery and categorizing total knee arthroplasty (TKA) candidates based on individual risk factors [25-33].

AI/ML models can also help predict and manage postoperative complications, leading to improved patient outcomes through personalized care. Multiple studies reviewed demonstrate the accuracy of these models in forecasting various adverse events following surgery [25,34-36].

AI/ML algorithms have been used in several studies to model total knee and hip arthroplasty revisions, reoperations, and hospital readmissions. Postoperatively, these tools enable surgeons to forecast patient outcomes, including functional results and patient-reported outcome scores [14,37-43].

**Figure 1:**
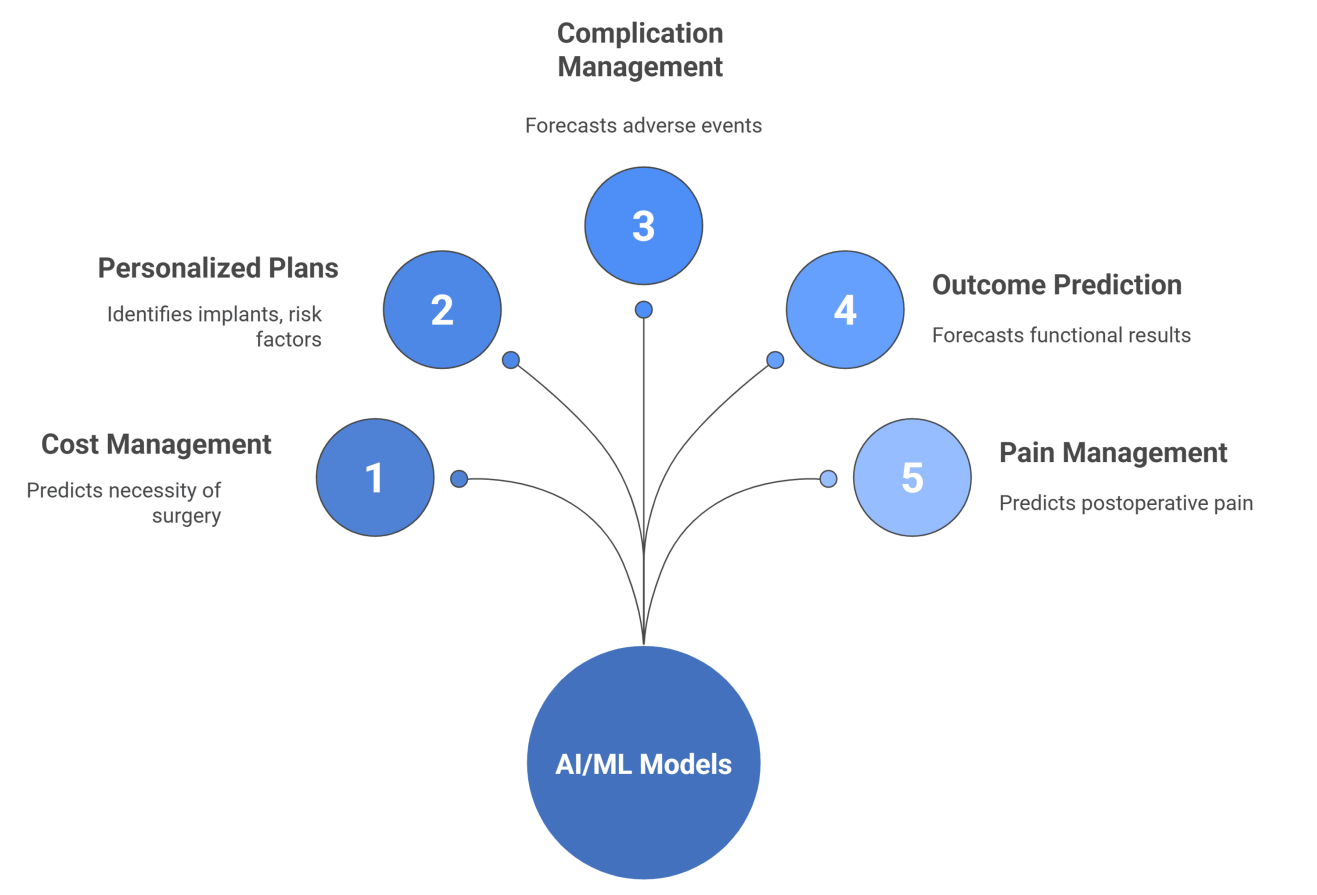
Overview of the Benefits of AI in Arthroplasty Data from [25-35]. This figure was created by the authors.

AI and ML technologies have also demonstrated the ability to accurately predict postoperative pain levels. This includes identifying patients at elevated risk of requiring prolonged opioid prescriptions after surgery. The insights gained from these AI and ML tools can enhance the development and implementation of analgesic and pain management protocols, particularly with respect to judicious opioid prescribing. Furthermore, these tools enable surgeons to further individualize treatment planning, which may even support consideration of nonoperative management options in selected cases, especially when a high predicted risk of revision surgery is identified (Figure 1) [42-45].

Preoperative planning

As the population ages, the incidence of hip disease and fragility fractures is rising. THA stands out as one of the most effective treatments for older patients [46,47]. A wide array of implant types is currently available, and the incidence of revision surgeries for these implants is unfortunately on an upward trend [48,49].

For a revision surgery to be successful, it is crucial to prepare the device by gathering details about previous implants, such as their size and type. Given the increase in revision patients over the last three decades, many patients may not have access to information about their prior surgeries [50].

Identifying the specific implant design before performing revision total joint arthroplasty is essential. A study by Wilson et al. indicated that orthopedic surgeons’ primary method for identifying failed hip and knee components pre-surgery is patient X-rays, supplemented by hospital operative records, office records, operative dictation reports, and implant sheets or labels [51]. Surgeons and their teams spent an average of 20-30 minutes per case identifying devices, totaling approximately 41 hours annually per surgeon. The projected opportunity cost related to this could reach $27.4 million by 2030, calculated based on Medicare reimbursement rates for surgeon evaluation and management services [52]. Surgeons struggled to identify the device before surgery in about 10% of cases and during surgery in approximately 2% of cases. This failure to identify the device beforehand resulted in negative consequences such as bringing at least two additional implants into surgery, longer operating room times, more complex surgeries, increased blood loss, greater bone loss, extended recovery times, and higher healthcare expenses [51,52].

Borjali et al. developed a novel, automated approach using a deep convolutional neural network (CNN) to identify the design of total hip replacement (THR) implants from plain radiographs. This addresses a significant clinical challenge, as manual identification of failed implants for revision surgery is time-consuming and error-prone, and failure to identify them can lead to increased surgical time, blood loss, and healthcare costs. This retrospective study used 252 AP hip X-rays of three common implant designs: Accolade II, Corail, and S-ROM. The authors compared a CNN retrained specifically on the X-rays with a pretrained CNN that used weights from the nonmedical ImageNet database. The retrained CNN, aided by online data augmentation, achieved 100% accuracy on the test subset. Conversely, the pretrained CNN failed to learn the task, suggesting that features from nonmedical images may not transfer well to this specific orthopedic application. Saliency maps confirmed that the retrained CNN focused on relevant features such as the stem tip, collar, and metaphyseal sleeve. The authors concluded that this deep learning method can accurately identify THR implants in seconds, potentially improving patient outcomes and reducing costs [26].

Kang et al. developed and validated a machine-learning pipeline to identify femoral stem designs on postoperative anteroposterior hip radiographs. They assembled 170 X-rays spanning 29 implant brands from five hospitals and web sources, applied preprocessing and heavy augmentation (3,606 stem crops), and built a two-stage model: YOLOv3 for stem detection followed by a CNN classifier for brand recognition. Using a 75/25 split, the system detected stems with a mean average precision >0.99 and achieved a mean ROC-AUC (receiver operating characteristic area under the curve) of 0.99 for brand classification on held-out images. The authors propose clinical uses in revision planning, surveillance for osteolysis or periprosthetic fracture, and automated registry curation, while noting dataset size and single-view limitations, indicating high feasibility pending larger validation and generalizability across centers [30].

Jang et al. developed a deep-learning workflow to estimate the hip joint center (HJC) on anteroposterior pelvis radiographs to support total hip arthroplasty (THA) planning. Using 3,965 patients (7,930 hips) from the Osteoarthritis Initiative, a U-Net segmented key bony landmarks, and pelvic-height ratios were optimized via grid search to predict HJC. Three models were evaluated: nonspecific, sex-specific, and patient-specific (using the contralateral hip). On an independent test set (1,262 hips), the system processed images at ~0.65 seconds per hip and predicted HJC within 5 mm in 80%, 83%, and 91% of cases. Median error fell to 2.82 mm with the patient-specific approach. Landmark segmentation achieved a Dice coefficient of 0.91. The authors note 2D imaging and the need for external validation as limitations; nevertheless, the tool may reduce variability and improve preoperative planning [53].

Postoperative outcome prediction

Several authors have demonstrated the potential ability of ML to predict patient outcomes following arthroplasty, showcasing its promise in enhancing patient care and surgical planning [14,43,54-56]. Predicting outcomes can aid orthopedic surgeons and patients in making shared decisions, especially regarding whether procedures will align with patient expectations [43,56]. Identifying patients who are unlikely to experience significant improvements in postoperative patient-reported outcome measures (PROMs) early on could lead to more intensive postoperative monitoring and improved surgical decision-making [14].

Multiple studies have investigated the minimal clinically important difference (MCID) after surgery, which signifies the degree of improvement in PROMs that a patient deems valuable. Fontana et al. demonstrated that supervised ML algorithms could predict two-year postsurgical MCID for general and joint-specific health PROMs with fair to good accuracy [14]. More recent research by Kunze et al. and Harris et al. demonstrated that ML could predict the MCID for patients undergoing THA and TKA, respectively. This capability could aid in optimizing preoperative health, improving patient selection, and enhancing patient education and satisfaction [55,56].

Postoperative patient satisfaction is another metric used to assess an intervention’s effectiveness. Studies by Farooq et al. and Kunze et al. both found that ML algorithms were more accurate than statistical or standard logistic regression models in predicting patient dissatisfaction after TKA. Accurately predicting dissatisfaction could enable better preoperative health optimization and improved patient-doctor communication [54,57].

Shohat and colleagues utilized ML to aid in treatment decisions for acute prosthetic joint infection (PJI). They created an algorithm that could accurately predict the success of debridement, antibiotics, and implant retention (DAIR) procedures, which typically have low morbidity but unpredictable outcomes, by considering the patient’s clinical presentation, comorbidities, physical examination, and laboratory results [58].

Gabriel et al. developed a predictive model to identify elective THA patients unlikely to require prolonged length of stay (LOS ≤3 days). In a single-institution retrospective cohort (2014-2016), multivariable logistic regression produced a point-based calculator using nine variables: age, opioid use, METs, sex, anemia, chronic obstructive pulmonary disease, hypertension, obesity, and primary anesthesia type. Performance on the validation set showed AUC 0.735 (95% CI 0.675-0.787) with good calibration (Hosmer-Lemeshow P = 0.37). At a score threshold of 12, the positive predictive value was 86.1%. Models using ridge, LASSO, and random forest were also tested. The tool may improve bed planning and resource utilization [59].

Sniderman et al. investigated patient factors predicting outcomes after THA using a machine-learning LASSO model. In a prospective cohort of 160 patients with degenerative arthritis, preoperative and three-month self-reports informed four predictor domains: demographics, patient-reported health, cognitive appraisal, and surgical approach. The model predicted three-month HOOS scores, finding cognitive appraisal variables most influential. Worse outcomes were associated with frequent work-related thoughts, social comparison to healthier peers, higher BMI, more comorbidities, and the anterior approach. Better outcomes were linked to being employed and thoughts emphasizing family interaction, trying not to complain, and helping others. These findings highlight that mindset and appraisal processes shape functional recovery after THA, suggesting value in preoperative counseling and targeted psychosocial interventions to optimize results [60].

Nemes et al. developed and validated a shared decision-making tool to predict one-year EuroQol five-dimension (EQ-5D) outcomes after total hip replacement using the Swedish Hip Arthroplasty Register. Models were trained on 2008 data and tested on 2009-2012 to assess temporal validity. Eligible cases were primary osteoarthritis with preoperative PROMs; exclusions included reoperation or death within one year, BMI >45, and resurfacing procedures. Predictors were preoperative EQ-5D (index and dimensions), EuroQol visual analogue scale (EQ-VAS), pain visual analogue scale (VAS), age, sex, Charnley class, American Society of Anesthesiologists physical status classification (ASA), BMI, implant type, and surgical approach. Two strategies were tested: direct regression of the EQ-5D index and classification of the five EQ-5D dimensions weighted by the United Kingdom time trade-off (UK TTO) value set. Models explained approximately 17% of the variance and remained stable over time, but predictions for severe states were poor; simple linear regression performed nearly as well as more complex methods [61].

Karhade et al. developed machine-learning models to preoperatively predict sustained opioid prescribing ≥90 days after THA. In a multicenter cohort (5,507 osteoarthritis cases, 2000-2018), 6.3% experienced prolonged postoperative prescriptions. Five algorithms were trained and evaluated for discrimination, calibration, and utility; elastic-net penalized logistic regression performed best (c-statistic 0.77). Key predictors included age, duration of prior opioid exposure, preoperative hemoglobin, and medications (antidepressants, benzodiazepines, nonsteroidal anti-inflammatory drugs (NSAIDs), beta-2-agonists). The final model was deployed as an explainable web calculator. If externally validated, it could meaningfully improve preoperative risk stratification and targeted support [45].

Bini et al. conducted a prospective study to test whether ML on wearable patient-generated data can predict six-week patient-reported outcomes after total joint arthroplasty. Twenty-two patients were enrolled, and fifteen completed the study, generating approximately 3 million data points from three trackers across 35 features spanning four weeks preoperatively to six weeks postoperatively. PROMs included the Hip and Knee Disability and Osteoarthritis Outcome Score, Knee Osteoarthritis Outcome Score, and VR-12 Physical Component Score at baseline and six weeks. Models identified three feature sets (A, B, C) most correlated with PROMs; data through postoperative day 11 yielded the highest predictiveness. Unsupervised clustering assigned patients to three PROM clusters, with a total sum of squares ranging from 3.86 (A) to 1.86 (C). These findings support early ML-based surgical risk stratification [62].

**Figure 2:**
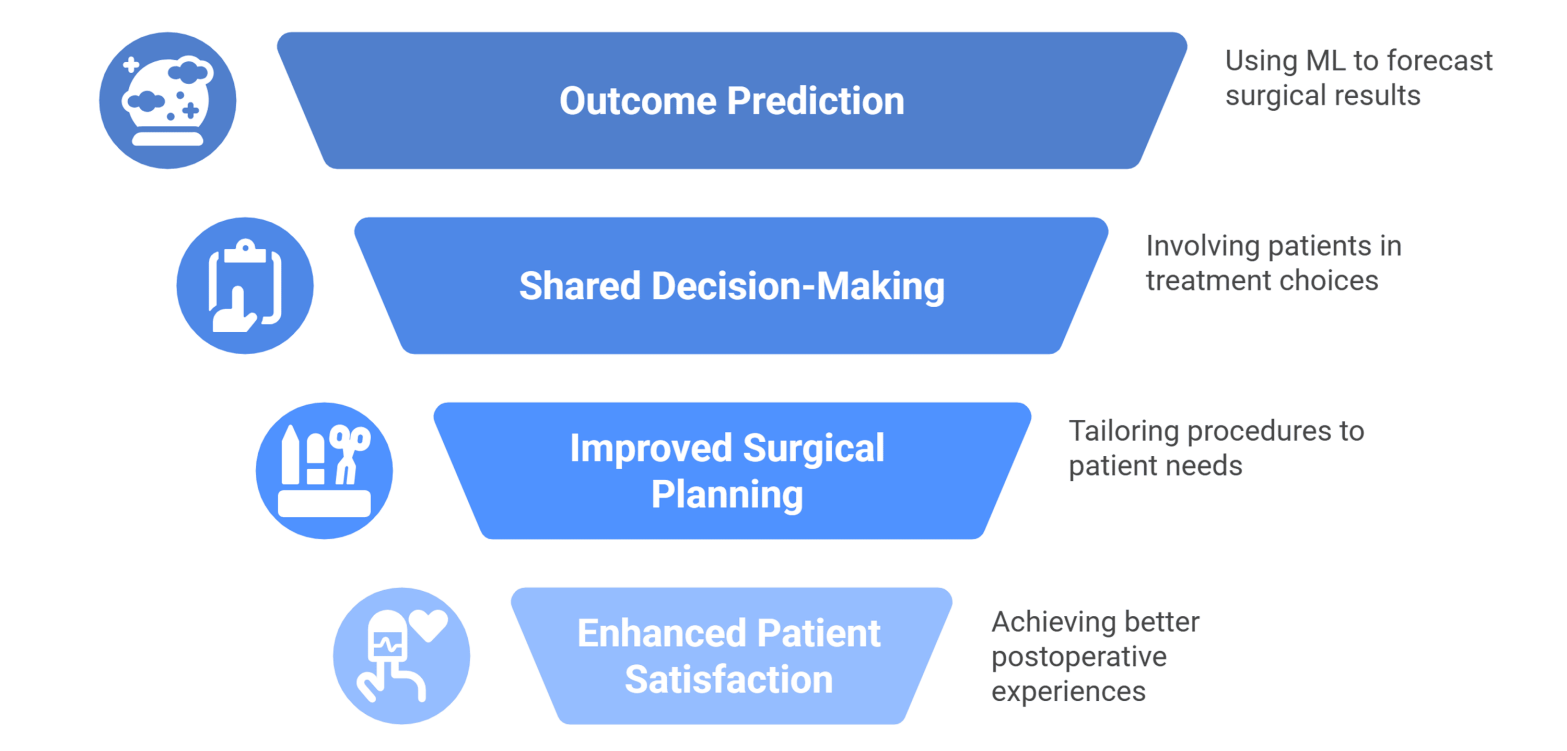
Enhancing Arthroplasty Outcomes With Machine Learning Data from [54-62]. This figure was created by the authors.

El-Galaly et al. used the Danish Knee Arthroplasty Registry to test whether machine-learning models can predict early revision within two years after primary TKA. They trained four models, i.e., LASSO logistic regression, random forest, gradient boosting, and a neural network, on 25,104 cases from 2012-2015 and temporally validated them on 6,170 TKAs performed in 2016. Age, postfracture osteoarthritis, and weight emerged as the most informative preoperative features; however, all models performed poorly (validation AUC 0.57-0.60), no better than a noninformative baseline. The authors concluded that preoperative registry variables alone cannot yield clinically useful predictions and that adding comorbidities, surgeon identifiers, and intraoperative and postoperative factors may meaningfully improve discrimination (Figure 2) [21].

Limitations and future directions

Most existing models have yet to undergo the rigorous testing and external validation needed to determine whether they work reliably in real-world clinical practice [40]. Without these evaluations, their performance, generalizability, and safety remain uncertain, limiting confidence in their effectiveness and preventing responsible deployment across diverse patient populations and clinical settings.

The primary benefit of AI in arthroplasty care appears to revolve around the optimization of patient outcomes through data-driven approaches [19]. The technology has already demonstrated significant value in supporting and driving clinical decision-making processes. Furthermore, the continuous incorporation of PROMs into a feedback loop consistently contributes to improvements in both subjective patient satisfaction and objective clinical outcomes [16,23,63,64].

Numerous large studies have been conducted after hip and knee replacement surgeries to determine the smallest clinically significant differences in various common PROMs. This helps to better distinguish between "statistical" and "actual" clinical improvements, a distinction that is expected to become more precise with the ongoing advancement of sophisticated AI algorithms [16,29].

As medical practices increasingly adopt technology for data management, including AI, there is an opportunity to standardize how patient data are collected and stored. This would enable easier integration of electronic medical records with patient-generated health data systems, allowing for improved data analysis. However, current inconsistencies in computing platforms and operating systems hinder this progress. While open-access platforms would improve data usability, they currently lack the necessary privacy safeguards for widespread healthcare use. Therefore, further development is needed to ensure that patient privacy and rights are protected as technology continues to advance [24,26].

## Conclusions

AI has demonstrated significant value in supporting and driving clinical decision-making across the entire arthroplasty care pathway. The primary benefit revolves around the optimization of patient outcomes through data-driven approaches. This includes enhancing preoperative planning by accurately identifying implant designs on radiographs, assisting in patient selection and risk stratification, and predicting postoperative trajectories. By forecasting complications, patient-reported outcomes, dissatisfaction, and even prolonged opioid use, AI enables earlier, more personalized interventions. Integrating AI with clinical and imaging data helps streamline decisions and create tailored care pathways, which can reduce time and costs for a typically older, comorbid patient population.

Despite these promising results, widespread adoption of AI in routine arthroplasty care requires overcoming significant challenges. Most existing models have yet to undergo the rigorous, large-scale external validation needed to ensure they are safe, reliable, and transportable across diverse clinical settings and patient populations. To translate these promising tools into reliable and equitable clinical practice, there is a critical need for stronger multi-center studies, transparent performance reporting, and reproducible evaluation standards. Future progress also hinges on establishing robust data standards and governance to overcome inconsistencies in computing platforms and ensure patient privacy is protected as data integration advances.
